# Effect of Hydrated Lime on Indirect Tensile Stiffness Modulus of Asphalt Concrete Produced in Half-Warm Mix Technology

**DOI:** 10.3390/ma13214731

**Published:** 2020-10-23

**Authors:** Mateusz M. Iwański

**Affiliations:** Department of Building Engineering Technologies and Organization, Faculty of Civil Engineering and Architecture, Kielce University of Technology, Al. Tysiąclecia Państwa Polskiego 7, 25-314 Kielce, Poland; matiwanski@tu.kielce.pl

**Keywords:** asphalt concrete, foamed bitumen, half-warm mix asphalt (HWMA), hydrated lime, indirect tensile stiffness modulus

## Abstract

Half-warm mix asphalt (HWMA) mixtures can be produced at temperatures ranging from 100 °C to 130 °C, depending on the production methods used. The lowest mixing temperature can be achieved by using water-foamed bitumen. The mixture should be characterized by a long service life, defined by the resistance to permanent deformation and high stiffness modulus at temperatures above zero. It is therefore important to ensure the adequately high quality of the bitumen binder. Bitumen 50/70 was provided with appropriate quality foaming characteristics (expansion ratio, ER, half-life, t1/2) by adding a surface-active agent (SAA) at 0.6 wt % before foaming. Then asphalt concrete (AC) 8 S was designed and produced with the recommended water-foamed binder. Hydrated lime, an additive substantially affecting asphalt concrete mechanical parameters, was used at 0, 15, 30, and 45 wt % as a partial replacement for the limestone filler. The influence of the amount of hydrated lime on the content of voids, indirect tensile stiffness modulus at −10 °C, 0 °C, +10 °C, +20 °C, and +30 °C, and the resistance to permanent deformation was investigated. Statistical analysis of the test results showed the quantity of 30% to be the optimum hydrated lime content. The AC 8 S resistance to permanent deformation was determined at the optimum hydrated lime content. The comprehensive evaluation revealed a synergistic effect between bitumen 50/70, modified before foaming with 0.6 wt % SAA and 30 wt % hydrated lime as the limestone filler replacement, and the half warm mixture AC 8 S, in terms of the standard requirements and durability of the HWMA concrete in pavement applications.

## 1. Introduction

Half warm mix asphalt (HWMA) comprises the most environmentally friendly technologies of bituminous mixture production with water-foamed bitumen used as a binder [1,2,3]. Mixtures with foamed binders can be produced at temperatures 60 °C lower than the conventional mixture temperatures. Both HWMA and hot mix asphalt (HMA) are subject to the same performance criteria, with key characteristics being high stiffness modulus at temperatures above zero, and high resistance to permanent deformation.

A high level of bitumen foaming parameters (expansion ratio ER, half-life t_1/2_) ensures proper mixture quality. As no formal requirements for the foaming parameters of water-foamed bitumen exist, the only applicable guidelines are those designed for cold recycling [4,5]. The properties of recycled mixtures with water-foamed bitumen have been widely investigated [6,7,8,9]. Several researchers have found that zeolite can be used to foam bitumen and bituminous mixtures instead of water, but this solution is used primarily with warm mix asphalt (WMA) [10,11,12,13].

The proper quality of the foam is ensured by adding various additives to the bitumen before foaming. Fischer–Tropsch (F–T) synthetic wax is the most commonly used additive [14,15,16]. Additives are also incorporated in bituminous mixtures made with foamed bitumen [14].

Following the modification, the bitumen foaming parameters (ER, t_1/2_) show markedly higher values compared with non-modified binders [17,18]. Adding F–T wax improves the standard and rheological properties of bitumen [19] and bituminous mixtures, especially in terms of deformation resistance [20,21]. The drawback of F–T wax addition is its crystallization at temperatures below 90 °C [22]. As a result, at low temperatures, compaction is difficult, and the mixture fails to attain the required material parameters [23].

A fundamental issue concerning low temperature bituminous mixtures is securing proper compaction. As the quality of compaction may depend on the additive used, new types of chemical additives are being designed, with a view to improving mixture compaction at reduced temperatures [24,25]. Although effective in increasing mechanical characteristics, some of the additives reduce moisture resistance [25]. Various chemical additives have been studied in the process of investigating the effect of compaction on mixture parameters. It was found that both the type of additive and the method of compaction significantly affected mix properties. However, the test results obtained varied with the additive type and compaction method used, for example, the results from the gyratory press were found to be more beneficial than those from the traditional Marshall method [26]. A decline in the mixture parameters was recorded with the lowering of compaction temperature, except for the case when F–T synthetic wax was used. In another study [19], both compaction methods provided traditional mixtures with comparable properties in terms of resistance to permanent deformation, but the stiffness modulus and tensile strength decreased. Extensive comparative tests with chemical, organic, and zeolite additives [27] showed a significant effect of mixing and compaction temperatures on the gyratory compaction and mechanical properties, such as sensitivity to water and stiffness modulus. When the temperature was lowered, mixtures with additives required less compaction energy, their susceptibility to moisture improved in comparison with the control mixture, and the test temperature had a significant effect on the stiffness modulus.

As shown above, there is no clear-cut, unambiguous assessment of the influence of the additive type and compaction method on the properties of bituminous mixtures. Each case of additive application, as well as the choice of the compaction method, must be considered individually.

It is therefore necessary to continue the study in search of an additive that will ensure proper bitumen foaming, without adversely affecting the properties of bituminous mixtures at the service temperature of about 90 °C. The surface-active agent (SAA), which reduces bitumen viscosity, [24,28] seems to be such an additive.

Exploratory research carried out in this respect confirmed a considerable influence of this additive on the bitumen foaming characteristics [29]. The studies and implementation practice to date show that SAA significantly improves the binder—aggregate adhesion in HMA, thus providing it with the required moisture and frost resistance. However, no improvement of bitumen properties has been observed after SAA incorporation. In some cases, a slight decline was reported [30]. Bituminous mixtures made with SAA-modified bitumen may exhibit less favorable properties than the mixtures containing non-modified bitumen [31]. Experience gained in HMA technology suggests that hydrated lime should be used in the research of foamed bitumen-based mixtures [32], since the lime has a substantial effect on the mix properties.

The use of hydrated lime as an additive to traditional HMA dates back over a hundred years [33,34].

Initially, the studies of hydrated lime addition focused on its influence on the adhesion between the mineral mixture and the binder. The positive results of those studies allowed expanding the scope of interest. It was confirmed that hydrated lime increased the resistance of the mixture to moisture and frost [35,36] as a result of improved adhesion of the bitumen to aggregate particles [37]. Its use is particularly important when using acidic aggregate (with SiO_2_ content >65%). The interaction mechanism is that calcium cations bond with silica compounds on the surface of the acidic aggregate, which results in the formation of strong ionic bonds.

It has been established that the mechanism of chemical interaction of hydrated lime on bitumen involves, first neutralizing bitumen polar molecules, and then their partial adsorption by hydrated lime molecules [38,39]. The remaining polar molecules of the bitumen, neutralized by the hydrated lime, do not diffuse to the bitumen–aggregate interface, but remain in the binder layer. The adhesion of the bituminous film to aggregates improves as the binder at the phase interface is neutral or alkaline [40,41,42].

Hydrated lime has been demonstrated to contribute to slowing down the rate of bitumen ageing, and hence the ageing of bituminous mixtures and pavements. This effect contributes to ensuring the durability of pavements throughout their service life [38,43].

The testing methodology includes two approaches. The first one is a laboratory-based approach in which bitumen is combined with hydrated lime or quicklime. The blend is then cured for a certain time, and then the lime is separated from the bitumen by extraction. The properties of the bitumen are determined at the end of this process [44,45,46,47,48]. The second method consists of testing bitumen recovered from pavement structural layers that were made of a bituminous mixture containing hydrated lime [49,50,51].

Tests of the binder recovered from bituminous mixtures used in the construction of structural layers are of particular importance in identifying the influence of hydrated lime on bitumen properties. It was found that the recovered binder exhibited higher viscosity than the binder that did not contain any surfactants [44]. A large number of studies have confirmed the positive role of hydrated lime in reducing the rate of changes in the properties of bitumen exposed to high temperatures during mixture production [52,53,54,55,56,57].

Hydrated lime is known to substantially increase the stiffness of the mastic defined by an increased softening point [58,59]. This parameter plays a very important role in ensuring the resistance of bituminous mixtures to permanent deformation. Increased mastic stiffness leads to increased rutting resistance. At the same time, excessive stiffness of the mastic may decrease the resistance of the bituminous mixture to fatigue and thermal cracking [60]. For these reasons, the amount of hydrated lime used needs to be limited and determined empirically. As such, hydrated lime can be used to control the stiffness of the mastic.

Hydrated lime has a positive effect on the mechanical characteristics of bituminous mixtures [61,62]. The dynamic modulus of bituminous mixtures, studied in the temperature range from −10 °C to +54.4 °C and at a frequency from 0.1 Hz to 25 Hz, increased by 8% to 65%, depending on the amount of hydrated lime dosed.

The influence of hydrated lime on the mechanical properties of bituminous mixtures has been studied extensively. Comprehensive research programs confirmed that the rut resistance of the mixture increases with increasing lime concentration, in the range from 2 wt % to 5 wt %, or with its quantity up to 30%, as a replacement of mineral filler in the mixture [46,63,64].

Resistance to permanent deformation has been studied for the dosing method, which was found to strongly impact the intensity of the effects on the mixture properties [35].

Attempts to use hydrated lime have also been made in the production of WMA mixtures. The overall results of those studies revealed that hydrated lime contributes to ensuring resistance to moisture and frost [65,66]. However, there is limited experience in this area. The same effect has been confirmed by the exploratory studies of hydrated lime in HWMA concrete. Further research in this respect is needed [67].

To sum up, the problem of searching for a bitumen additive that allows lowering the mixing temperature of the bituminous mixture is still valid, especially when it is produced with water-foamed bitumen at about 100 °C. Surface-agent additives are used to improve foaming parameters of the binder, and to secure the high quality of the bituminous mixture. The durability of the mixture is ensured by the use of hydrated lime.

The beneficial effect of hydrated lime on a wide range of the properties of traditional HMA mixtures has already been confirmed. There is reason to believe that the same effects, that is, high values of the stiffness modulus and improved resistance to permanent deformation, will be confirmed for HWMA mixtures with water-foamed bitumen.

## 2. Methodology

### 2.1. Material

The laboratory test material was HWMA concrete AC 8 S produced with 5.6% foamed bitumen 50/70, following the relevant requirements [68]. For reference purposes, the amount of binder was increased to 5.9%, 6.2%, and 6.5%. In this way, the increased demand for the binder, due to the use of hydrated lime in the asphalt concrete, was taken into account [28].

The bitumen used it the tests was a 50/70 paving grade bitumen, commonly used in the countries of central and eastern Europe in mixtures designed for pavements, under traffic characterized by 2.5 × 10^6^ < ESAL_100 kN_ < 7.3 × 10^6^ (ESAL: equivalent single axle load) [69]. In Poland, it is the highest penetration bitumen that is permitted for use in bituminous mixtures designed for pavement wearing course [68]. The use of softer bitumen is not allowed due to the risk of permanent deformation.

Before foaming, the bitumen was modified with a 0.6% fatty acid amide-based surface active agent (SAA), by weight of the binder, to obtain high values of foaming characteristics. The properties of the SAA were as follows:

- appearance: brown viscous liquid,
- density at 20 °C: 0.98 Mg/m^3^,
- pour point: <0 °C,
- viscosity at 20 °C: 3000 mP,
- viscosity at 50 °C: 400 mP,
- amine index: 159–185 mg HCl/g,
- acid index: <10 mg KOH/g,
- freezing point: <0 °C,
- flash point (open flame): >218 °C.

The bitumen was water foamed. The foam expansion ratio (*ER)* [4,5] and half-life (t_1/2_) [4,5] were determined in the second stage of the study with 9 replicates [70,71].

Physical properties of the foam were tested in a Wirtgen WLB-10S foaming plant, by applying different foaming water amounts (FWC: foaming water content): 1.5 wt %, 2.0 wt %, 2.5 wt %, 3.0 wt %, and 3.5 wt %, as per [4]. The bitumen foaming test was conducted under the following conditions:

− bitumen temperature: 155 °C,
− water temperature: 20 °C,
− water flow: 100 g/s,
− foaming time: 5 s,
− air pressure: 500 kPa,
− water pressure: 600 kPa.

Results of the selected properties of the non-modified bitumen 50/70, and that modified with 0.6% SAA, are compiled in Table 1.

**Figure 1:**
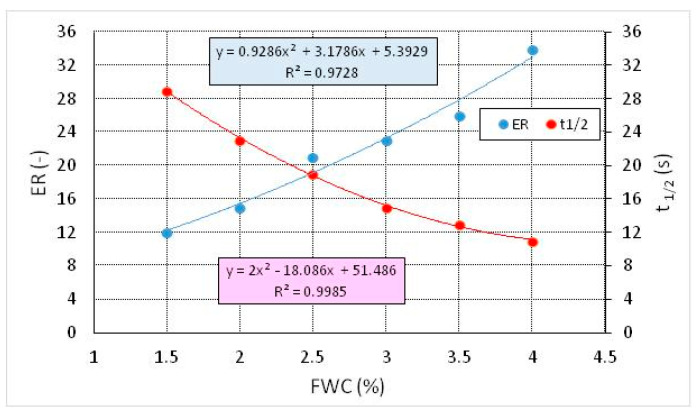
Foaming characteristics of bitumen 50/70 modified with 0.6% SAA [29].

Foaming characteristics of the bitumen modified with 0.6% SAA are shown in Figure 1.

The mineral mix of AC 8 S was designed using the aggregates commonly available in the Świętokrzyskie region: limestone filler, granulated limestone aggregate 0/2 mm, and granulated gabbro aggregate 2/5 mm and 4/8 mm.

The lime stone filler met all the requirements of EN 13043. The basic properties of the aggregate used in the asphalt concrete are compiled in Table 2 and Table 3.

The hydrated lime used in the tests met the requirements of EN-459-1 CL 90-s.

### 2.2. Mix Design and Sample Preparation

Bitumen 50/70 modified with 0.6% SAA is characterized by very high values of foaming parameters [25]. Thus, its use in the production of bituminous mixtures should ensure a very good coating of aggregate by the binder.

**Figure 2:**
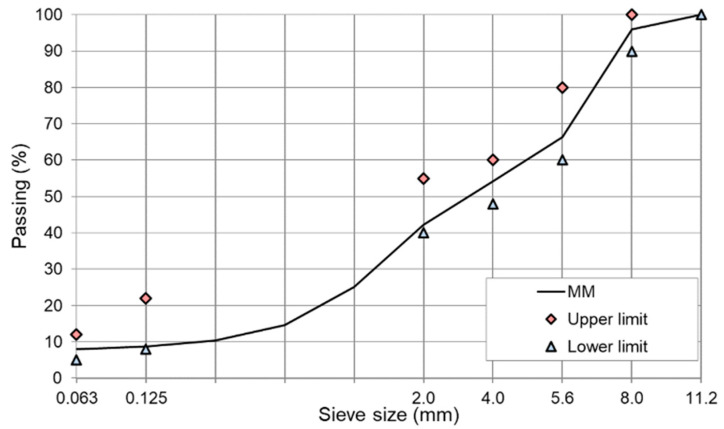
Grading curve of AC 8 mineral mixture with limiting points as in WT-2 2014 requirements [68].

The basic frame-compositions of the mineral mixture and bituminous mixture are summarized in Table 4 and the particle size design of AC 8 S is plotted in Figure 2 [68,72].

A variable bitumen amount was used in this study to determine its effect, in terms of hydrated lime dosing, on asphalt concrete properties. The grading of the aggregate mix was corrected while increasing the amount of foamed bitumen to 5.6% and 6.5%, according to the experimental design.

In order to ensure the required value of asphalt concrete parameters, hydrated lime was dosed at 15%, 30%, and 45% by weight as a replacement for an equivalent amount of lime filler.

The AC 8 S mixture was made in a heated mechanical mixer, to which foamed bitumen produced in the WLB-10S device was added. The production temperature of AC 8 S with additives did not exceed 100 °C.

**Figure 3:**
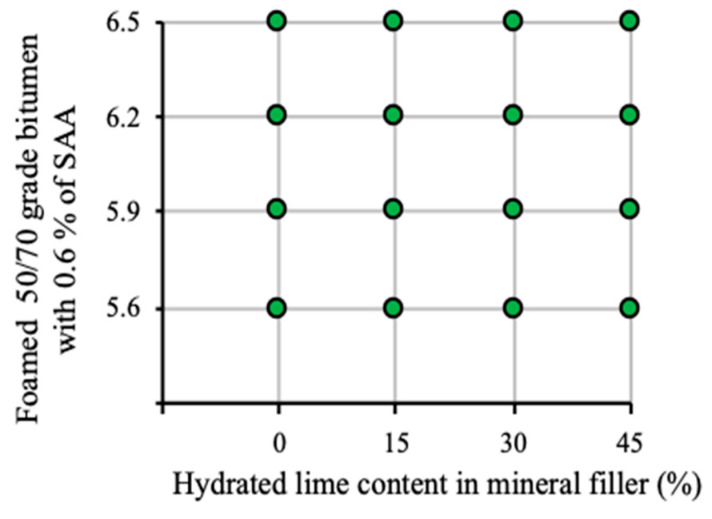
Experimental design [72].

The experimental design parameters of the AC 8 S were determined based on the assumed 4 × 4 factorial design, following the adopted research program (Figure 3).

### 2.3. Testing

This study aimed to determine the effect of hydrated lime on the properties of HWMA concrete with foamed bitumen, through the evaluation of the following parameters, and following the test procedures set out in the technical requirements of WT-2 2014 [68] and EN 13108-1,

- air void content (V_a_, %) as per EN 12697-8,
- indirect tensile stiffness modulus at −10 °C, 0 °C, +10 °C, +20 °C, +30 °C (S_m_, MPa), as per EN 12697-26,
- resistance to permanent deformation (WTS_AIR_, PRD_AIR_), as per EN 12697-22.

Parameters V_a_, and S_m_ were determined by compacting the specimens with a Marshall hammer, and using the number of blows as specified for each procedure used. The specimens (slabs) for testing the resistance to permanent deformation, parameters WTS_AIR_ and PRD_AIR_, were prepared with a dedicated compactor, as per EN 12697-22. The asphalt concrete (AC) specimens used met the assumed requirements, in terms of physical and geometrical characteristics.

#### 2.3.1. Air Void Content (V_a_)

The properties of asphalt concrete largely depend on air void content. An excessively high content of air voids adversely affects AC resistance to moisture and frost, and its performance under heavy loads. On the other hand, the insufficient content of air voids worsens the resistance of the bituminous mixture to permanent deformation, despite having a positive effect on moisture and frost resistance.

The percentage of air voids was calculated in accordance with EN 12697-8: 2005 on the basis of the following relationship

(1) $$V_{a}=\;\frac{\rho_{m\;}-\rho_{b}}{\rho_{m}}\;100\%$$

where: V_a_ is the air void content in the bituminous mixture (volume %); ρ_m_ is the theoretical maximum density of mixture (kg/m^3^); ρ_b_ is the bulk density of compacted mixture (kg/m^3^).

#### 2.3.2. Indirect Tensile Stiffness Modulus (S_m_)

The stiffness modulus of asphalt concrete S_m_ is a very important parameter, used for predicting AC behavior under different climatic conditions. In the range of winter negative temperatures, an increase in its value may indicate that the asphalt concrete is too rigid, which leads to the development of cracks and, consequently, to the destruction of the asphalt pavement. However, in the range of increasing summer temperatures, the parameter should reach the highest values, then the pavement will be resistant to permanent deformation. Stiffness modulus tests were performed at −10 °C, 0 °C, 10 °C, 20 °C, and 30 °C, corresponding to the average temperature of the winter period, spring transition period, the beginning of spring, the beginning of summer, and the average summer temperature.

**Figure 4:**
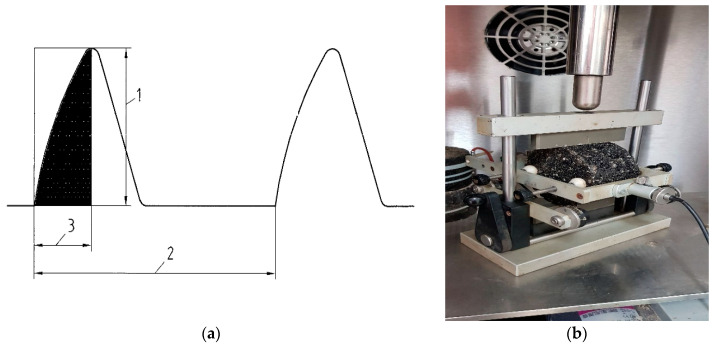
An example of a force applied to the specimen and the loading scheme [71]: (**a**) loading pulse form (1: maximum loading, 2: periodical force pulse, 3: force increase time), (**b**) set-up for indirect tensile stiffness modulus test; laboratory at Kielce University of Technology, (source: M. M. Iwański).

The stiffness modulus was determined with the standard load pulse applied to the specimen surface, following the procedure outlined in EN 12697-26 (Figure 4).

The test temperature of the mixture after the conditioning period was determined, with an accuracy of ±0.5 °C. To ensure the correct performance of the test, before the test, the appropriate value of Poisson’s ratio (dependent on the temperature and loading time) was entered into the calculation program. During the test, the load factor was read, and if it was 0.6, the test was correct.

The values of the stiffness modulus and Poisson’s ratio were determined from the following formulas:

(2) $$S_{m}=\frac{F\cdot \left(v+0.27\right)}{z\cdot h}$$

(3) $$v=3.59\cdot \frac{z}{∆V}\;-\;0.27$$

where:

S_m_ is the indirect tensile stiffness modulus [MPa],

F is the maximum force applied to the specimen [N],

ν is the temperature-dependent Poisson’s ratio,

z is the horizontal displacement amplitude for the specimen under loading [mm],

h is the specimen thickness [mm],

∆V is the maximum vertical displacement of the specimen (corresponding to maximum horizontal displacement) (mm).

Analysis of Equation (3) indicates that the increase in transverse strain and the specimen thickness contribute to the lowering of internal stresses in the pavement, and thus to the S_m_ reduction.

When the load factor was not 0.6, the stiffness modulus of the mixture was calculated using the following formula:

S_m_’ = S_m_∙[1 − 0.322∙(log (S_m_) − 1.82)∙(0.60 − k)] (4)

where:

S_m_’ is the stiffness modulus with the load factor accounted for [MPa],

k is the value of the load factor.

#### 2.3.3. Resistance to Permanent Deformation

Resistance to permanent deformation of the asphalt concrete was assessed to EN 12697-22. The test was carried out in air, in a small wheel tracking device, using rectangular prisms 400 mm × 300 mm and 40 mm thick, as outlined in WT-2 2014 [68]. The specimen mass varied with the density value obtained in the Marshall tests performed earlier. The testing procedure consisted of placing the specimen in a steel mold, and mounting it on a movable tray. After a 6-h conditioning period, starting from the moment the device reached the temperature of 60 ± 1 °C, the specimens were subjected to a load of 700 ± 10 N, using a wheel 200 mm in diameter with a tire 50 ± 1 mm in width, which tracked on the specimen along a length of 230 mm ± 10 mm. Rut depth was measured continuously on the specimen. The test wheel travelled 26.5 cycles per 60 s. The test was continued to reach 10,000 cycles. Results were used to determine the slope of the WTS_AIR_ rutting plot and the proportional rut depth (PRD_AIR_). The proportional rut depth and the maximum rut depth gain were calculated from:

(5) $${\mathrm{PRD}}_{\mathrm{AIR}}=\frac{{\mathrm{RD}}_{\mathrm{AIR}}}{h}\cdot 100\%$$

(6) $${\mathrm{WTS}}_{\mathrm{AIR}}=\frac{(d_{10000}-d_{5000)}}{5}\left[\mathrm{mm}/10000\mathrm{cycles}\right]$$

where:

RD_AIR_ is the rut depth (mm),

d_10000_ is the rut depth after 10,000 cycles (mm),

d_5000_ is the rut depth after 5000 cycles (mm),

h is the specimen height (mm).

For further analyses, the arithmetic mean of nine tests was taken as the final result of AC resistance to permanent deformation.

#### 2.3.4. Statistical Analysis of Test Results

The obtained test results were subjected to statistical analysis of variance (ANOVA), the purpose of which was to determine the significance of the impact of the given factor (hydrated lime, *HL*, foamed bitumen, *FB*) on the properties of the bituminous mixture [73,74].

The amounts of foamed bitumen and hydrated lime were assumed to be significant factors affecting the analyzed AC property when the *p*-value characterizing them was less than the assumed significance level α = 0.05.

The change in the value of the tested parameter (A) in asphalt concrete AC 8 S, relative to the contents of foamed bitumen 50/70 with 0.6% SAA and hydrated lime, was comprehensively described with a statistical model using the second-degree polynomial [73]:

(7) $$y=b_{0}+\sum_{i=1}^{n}b_{i}\cdot x_{i}+\sum_{i=j=1}^{n}b_{i=j}\cdot x_{i}\cdot x_{j}+\sum_{i=1}^{n}b_{ii}\cdot x_{i}^{2}$$

which in the performed analysis takes the form:

A = b_0_ + b_1_·x_1_ + b_2_·x_2_ + b_3_·x_1_·x_2_ + b_4_·x_1_^2^ + b_5_·x_2_^2^ (8)

where x_1_ = foamed bitumen (*FB)* (%), x_2_ = hydrated lime (*HL)* (%), b_0_-b_5_ are regression coefficients.

## 3. Results and Discussion

### 3.1. The Effects of Foamed Bitumen and Hydrated Lime on Air Void Content in Asphalt Concrete

Proper performance of the asphalt concrete in the pavement, and its durability, are secured when the air void content in the HWMA concrete is close to or more advantageous than that of the HMA concrete.

The air void content in AC 8 S was determined in accordance with the methodology set forth in EN 12697-6:2008. Asphalt concrete AC 8 S should have 2.0% to 4.0% air voids [68,69,72]. Each test series consisted of nine trials, the number of which was determined in accordance with [70,71]. The homogeneity of the results was confirmed by the coefficient of variation ranging from 3.36% to 7.77%. The ANOVA test showed that the content of foamed bitumen and hydrated lime was a significant factor that affected the quantity of air voids in AC 8 S, because the *p*-value was lower than the significance level α = 0.05. It was also found that there were interactions between the amount of foamed bitumen and hydrated lime, which influenced the air void content in the mixture (*p*-value less than α = 0.05).

**Figure 5:**
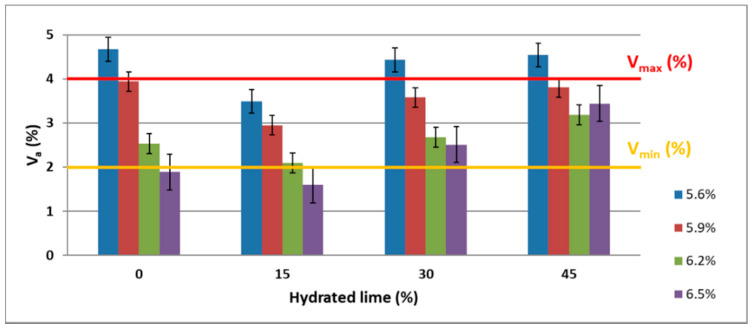
Air void content in AC 8 S as a function of the content of hydrated lime (0%, 15%, 30%, 45%) and foamed bitumen modified with 0.6% SAA (5.6%, 5.9%, 6.2%, 6.5%).

A graphical representation of the data is shown in Figure 5.

Analysis of the test results in Figure 5 leads to the conclusion that the content of both the foamed bitumen and the hydrated lime substantially affect the percentage content of air voids in AC 8 S. As the amount of foam bitumen increases to 6.5%, the air void content decreases, regardless of the hydrated lime quantity added. The use of hydrated lime at 15%, in place of a portion of limestone dust, alters this parameter considerably. The amount of air is reduced, which is certainly the effect of improving the adhesion between the bitumen and mineral mixture. The 30% and 45% m/m content of hydrated lime in place of limestone dust increases the air void content, regardless of the amount of foamed bitumen used in the asphalt concrete. This may be related to an insufficient amount of the binder, being the result of the use of hydrated lime, which has a larger specific surface area than limestone dust. As a result of the “deficiency” of the required amount of the binder, the mixture becomes difficult to compact to the proper level, and the air void content increases [32]. Regardless of the hydrated lime content, the smallest amount of air voids is found in AC 8 S containing 6.5% foamed bitumen. A high binder content, however, may cause it to fail to maintain adequate resistance to permanent deformation [30,31].

A comprehensive analysis of the air void content variation, with respect to the amount of hydrated lime and foamed bitumen 50/70 modified with 0.6% SAA, was presented using a statistical model [74].

Table 5 compiles the values describing the parameters of the regression model for the relationships between the air void content in AC 8 S and the content of SAA modified foamed bitumen and hydrated lime.

Analysis of the results (Table 5) indicates that both the quantity of foamed bitumen 50/70 and hydrated lime, as well as the interaction between these factors, have a substantial impact on the air void content in the asphalt concrete. A synergistic effect of the hydrated lime and foamed bitumen containing 0.6% SAA occurs in the asphalt concrete. The model of the analyzed relationships was adopted correctly because the adjusted coefficient of determination (R^2^) was 88%.

**Figure 6:**
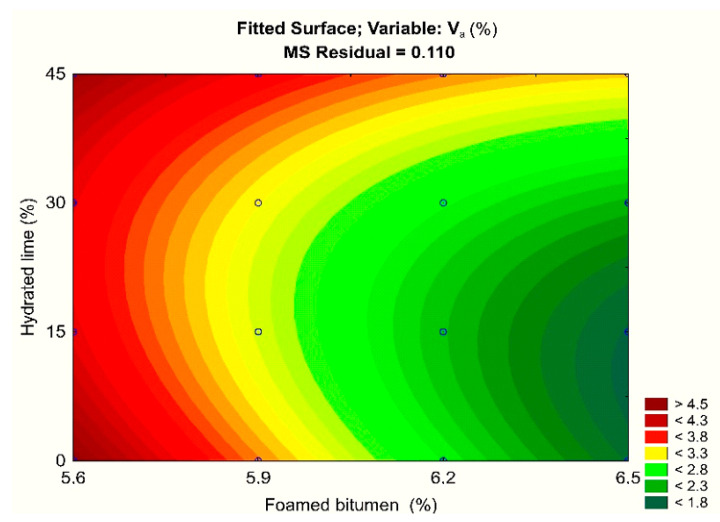
Air void content (V_a_) of AC 8 S as a function of the quantity of foamed bitumen and hydrated lime; the model describing these relationships V_a_ = 68.158 − 18.425FB + 1.266FB^2^ − 0.276HL + 0.001HL^2^ + 0.038FB∙HL [74].

The impact of hydrated lime and foamed asphalt on the content of air voids (V_a_) in AC 8 S for the developed model is shown in Figure 6.

The test results obtained show that, throughout the entire scope of the experiment, increasing the content of foamed bitumen and hydrated lime reduced the content of air voids in the asphalt concrete. At the same time, the hydrated lime had a significant effect on the assessed parameter; in the range from 5.6% to 5.9% of the foamed bitumen, it increased the air void content over the maximum recommended value of 4.0%. The increase in the foamed bitumen content in the range from 5.9% to 6.2%, at the hydrated lime content of 15% to 30%, allowed the parameter to achieve the recommended values [68]. A further increase in the foamed bitumen content up to 6.5% reduced the quantity of air voids below the required 4.0% [68].

### 3.2. Effect of Foamed Bitumen and Hydrated Lime on Asphalt Concrete Stiffness Modulus

A universal testing machine UTM-25 was employed to determine the stiffness modulus S_m_ of the asphalt concrete AC 8 S. Both the specimens and the accessories of the test device were placed in a thermostatic chamber prior to testing, as required by EN 12697-26. Each series of test specimens consisted of nine samples, the number of which was determined according to [70,71]. The results were characterized by high repeatability, as the coefficient of variation was between 2.17% and 8.80% throughout the entire experiment.

**Figure 7:**
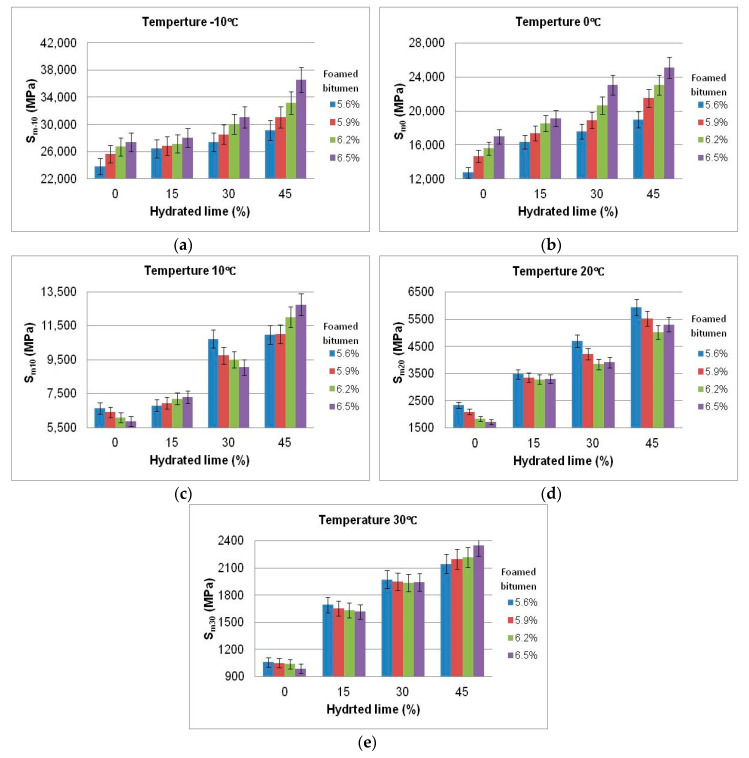
Stiffness modulus (S_m_) (IT-CY) of asphalt concrete AC 8 S, as a function of the contents of foamed bitumen and hydrated lime at various test temperatures, S_m-10_ (**a**), S_m0_ (**b**), S_m+10_ (**c**), S_m20_ (**d**), S_m30_ (**e**)

The results of stiffness modulus S_m_ testing at various test temperatures (−10 °C, 0 °C, 10 °C, 20 °C, and 30 °C) are shown in Figure 7.

The test results show that the content of hydrated lime has a significant effect on the stiffness modulus S_m_ of asphalt concrete AC 8 S at temperatures from −10 °C to 30 °C. The extent of this effect depends on the content of foamed bitumen. Test temperature also influences the value of this parameter. In the temperature range −10 °C to 0 °C, the influence of hydrated lime and foam bitumen is the same. Increasing the amount of both components causes the stiffness modulus to increase. Nevertheless, the effect of hydrated lime is more significant than that of the binder.

When increased from 10 °C to 20 °C, the test temperature alters the nature of the interaction between hydrated lime and foamed bitumen. The effect of the binder becomes more important, although hydrated lime also plays a significant role.

A further increase in the test temperature up to 30 °C enhances the effect of hydrated lime on the modulus, while the amount of bitumen remains less significant. The observed relationship is very important, because it means that by substantially increasing the stiffness modulus, the hydrated lime provides asphalt concrete with greater resistance to permanent deformation.

The most beneficial effect of hydrated lime on the stiffness modulus of HWMA concrete AC 8 S, in the test temperature range from −10 °C to +30 °C, can be observed for the HL content from 15% to 30%, and foamed bitumen content from 5.9% to 6.2%. Proper performance of asphalt concrete AC 8 S in the structural layer of the pavement will be ensured, both in winter (no cracks) and in summer (resistance to permanent deformation).

A second-degree polynomial model was adopted to comprehensively describe the relationship between the stiffness modulus (S_m_) of HWMA concrete AC 8 S (foamed bitumen 50/70 modified with 0.6% SAA; hydrated lime) and the test temperature. The model was assessed with the analysis of variance (ANOVA) [73,74].

Analysis of the parameters indicates that the amount of foamed bitumen and hydrated lime is a significant factor that affects the stiffness modulus (S_m_) of the asphalt concrete AC 8 S, because the levels of *p*-value related to them are lower than the assumed significance level α = 0.05. The intensity of this effect relies on the test temperature, and is most pronounced at temperatures −10 °C, 20 °C, and 30 °C. Please note the significant role of the interactions between foamed bitumen and hydrated lime. The interactions affect the value of the parameter (the *p*-value is less than α = 0.05), thereby confirming the synergy between these two factors. This effect supports the assumption about the desirability of using both the foamed bitumen and the hydrated lime in asphalt concrete.

The value of the adjusted coefficient of determination (R^2^) ranges from 81% to 94%, depending on the stiffness modulus and the test temperature applied. The R^2^ values indicate the correctness of the adopted models for describing the relationships between the stiffness modulus (S_m_) of the asphalt concrete AC 8 S, and the amount of foamed bitumen and hydrated lime.

The parameters of the regression model for the relationship between the S_m_ and the amount of hydrated lime and foamed bitumen are summarized in Table 6.

Analysis of the parameters compiled in Table 5 confirms the significance of hydrated lime in affecting the stiffness modulus (S_m_) of AC 8 S, because the levels of *p*-value are lower than the assumed significance level α = 0.05. It is important to note, the interaction between the foamed bitumen and hydrated lime for the stiffness modulus, S_m-10_ and S_m10_ (the *p*-value is less than α = 0.05).

**Figure 8:**
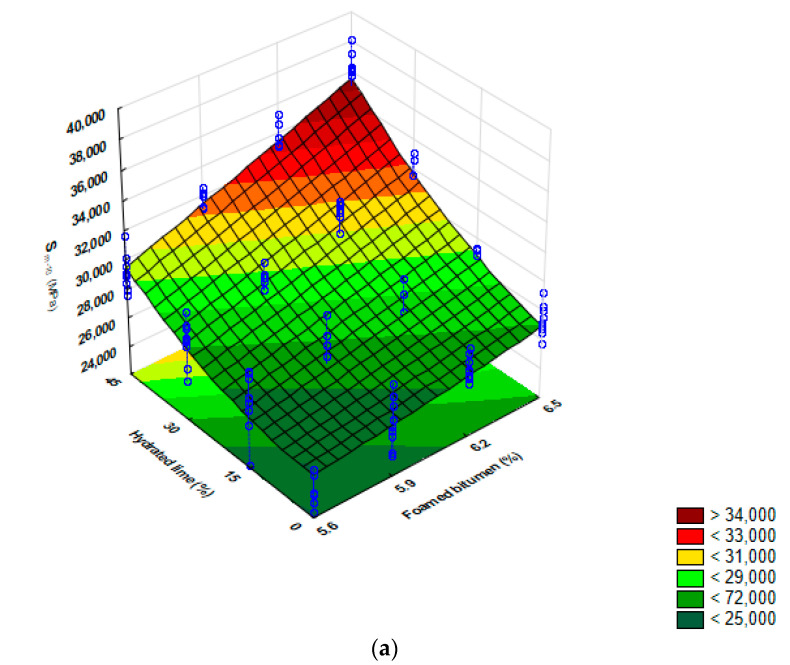

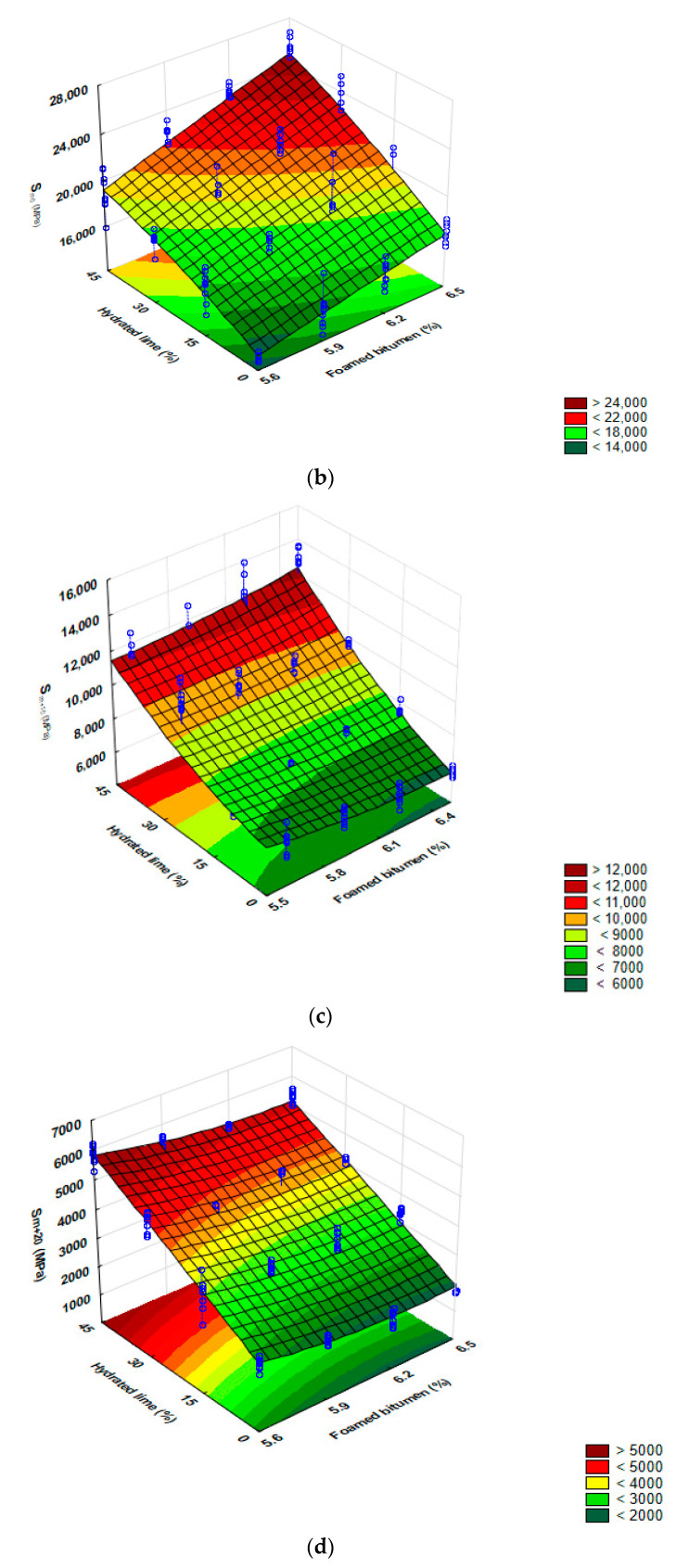

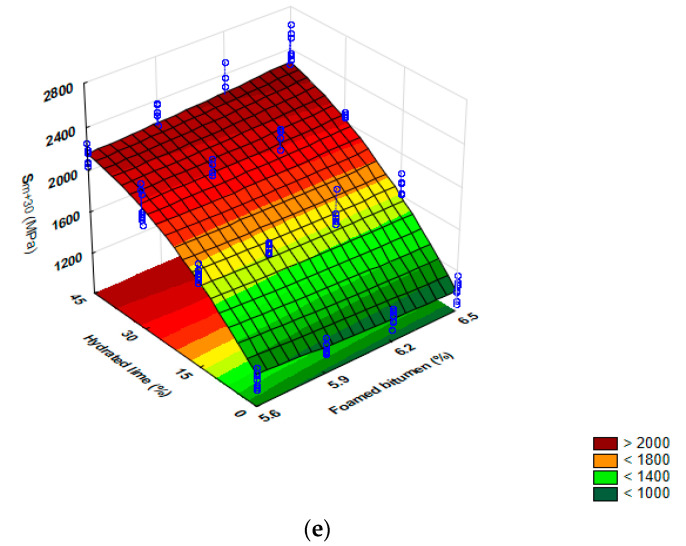
Stiffness modulus (S_m_) as a function of test temperature and amount of foamed bitumen and hydrated lime; models describing these relationships. (**a**) S_m-10_ = 31,976.2 − 4250.8 FB + 535.1 FB^2^ − 560.6 HL + 2.2 HL^2^ + 100.4 FB HL, (**b**) S_m0_ = - 14,876.7 +6178.5 FB − 203.2 FB^2^ − 178.1 HL − 0.8 HL^2^ + 61.4 FB HL, (**c**) S_m10_ = 42,843.7 − 11,206.5 FB + 846.5 FB^2^ − 194.2 HL + 1.2 HL^2^ + 43.8 FB HL, (**d**) S_m20_ = 44,108.5 − 13,433.1 FB + 1068.0 FB^2^ + 117.0 HL − 0.1 HL^2^ − 6.4 FB HL, (**e**) S_m30_ = 4073.4 − 872.1 FB + 61.3 FB^2^ + 6.5 HL − 0.4 HL^2^ + 6.0 FB HL.

The value of the adjusted coefficient of determination (R^2^) reaches nearly 81%, 85%, 87%, 94%, and 93%, respectively, at temperatures −10 °C, 0 °C, 10 °C, 20 °C, and 30 °C, which indicates a high reliability of the adopted models for describing the stiffness modulus (S_m_) against the amount of foamed bitumen and hydrated lime (Figure 8).

A comprehensive analysis of the results based on the data in Figure 8 indicates that at −10 °C, the value of the stiffness modulus (S_m-10_) shows no significant increase, with the amount of foamed bitumen increasing from 5.9% to 6.2%, and the amount of hydrated lime increasing from 15% to 30%. There is an interaction between the hydrated lime and foamed bitumen for providing AC 8 S with the resistance to low temperatures. The use of foamed bitumen in this quantity will have an advantageous effect on AC performance at low temperatures, making it less susceptible to low-temperature cracking than HMA.

In the entire domain of the experiment, an increase in the content of foamed bitumen and hydrated lime steadily increased the value of the stiffness modulus (S_m0_), with the highest increase rate occurring diagonally across the foamed bitumen/hydrated lime relationship. The hydrated lime interacts with foamed bitumen, and provides AC 8 S with the resistance to low temperatures. The most advantageous effect was observed for the hydrated lime used at 30%, as a replacement for the mineral filler.

A detailed analysis of S_m_ results within the test temperature range 10 °C to 30 °C shows that the amount of the hydrated lime, increasing from 15% to 45%, has a significant effect on the modulus value. At lower foamed bitumen content, the binder tends to interact with the hydrated lime, and this interaction increases the stiffness modulus of the AC 8 S mixture.

**Figure 9:**
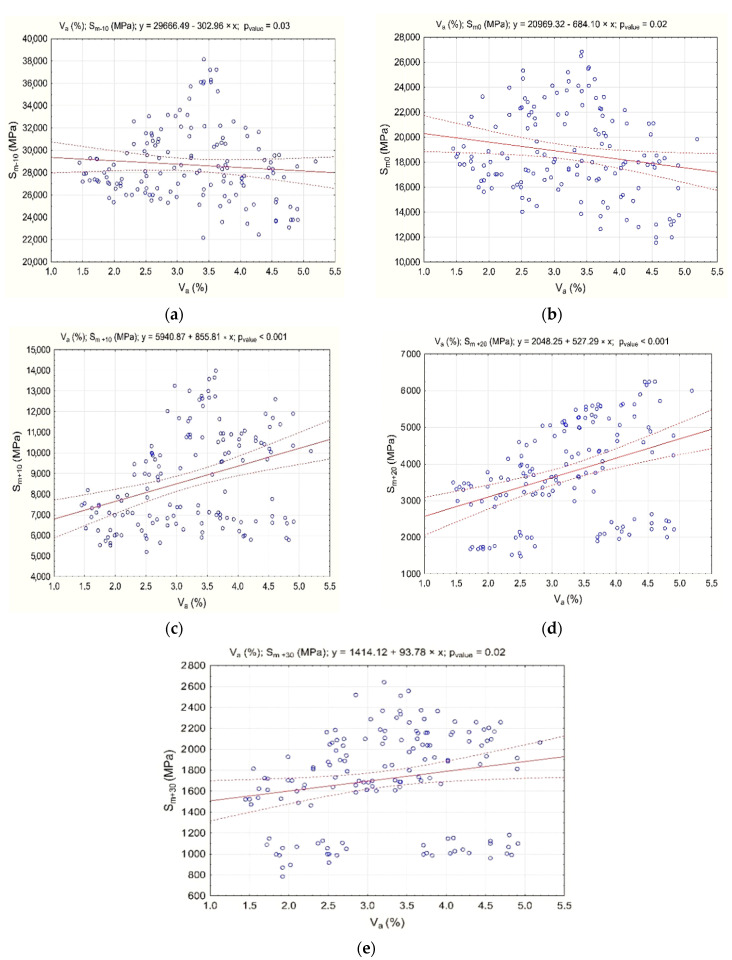
Correlation between AC 8 S mixture air void content (V_a_) and stiffness modulus S_m_; (**a**) S_m-10_, (**b**) S_m0_, (**c**) S_m+10_, (**d**) S_m+20_, (**e**) S_m+30_. Dotted lines represent 95% confidence interval.

To assess the effect of hydrated lime and foamed bitumen 50/70 with the addition of 0.6% SAA on the properties of the AC 8 S bituminous mixture, the correlation between the air void content (V_a_) and stiffness modulus (S_m_) was analyzed (Figure 9).

The statistically significant correlation between the air void content (V_a_) of AC 8 S and the stiffness modulus (S_m_) has a linear character (*p*-value less than α = 0.05).

### 3.3. Optimization of the Foamed Bitumen and Hydrated Lime Content in Terms of Service Durability of HWMA Concrete AC 8 S

An important element of the assessment of the impact of hydrated lime and foamed bitumen 50/70 modified with 0.6% SAA is the analysis of the relationships between the assessed properties of asphalt concrete AC 8 S. The analysis is summarized as correlations in Table 7 [74].

The main correlation parameter was the content of air voids V_a_, which had a significant impact on other properties of asphalt concrete AC 8 S. It can be concluded that the correlations between virtually all analyzed properties, except the stiffness modulus (S_m-10_) at −10 °C, are statistically significant, although not at the highest level. This effect may be related to the specific conditions (temperature −10 °C) of the test. The correlation values correspond to the analysis results obtained from the experiment, which shows that either most of the results are non-linear or statistically significant interactions occurred.

When assessing the impact of additives in both bitumen (50/70) and asphalt concrete mixtures (AC 8 S), it is very important to optimize them using the obtained mathematical regression models and statistical analysis program [73,74].

For ensuring the most favorable properties of AC 8 S, the recommended amount of hydrated lime and foamed bitumen was determined through the analysis of the results from a simultaneous response optimization of the following parameters (variables):

- air void content (V_a_) according to WT-2 2014 [68],
- stiffness modulus at −10 °C, 0 °C, +10 °C, +20 °C, and +30 °C, according to EN 12697-26, and depending on the content of the foamed bitumen and hydrated lime in HWMA concrete AC 8 S.

The models for the optimized variables are summarized in Table 8.

The relationships described above and the evaluation of the values listed in Table 7 show that all analyzed models adequately describe the most important properties of AC 8 S, and can be used to optimize its composition. The adjusted determination coefficient (R^2^) assumes values from 0.789 to 0.942, which indicates a high reliability of the optimization parameters.

**Figure 10:**
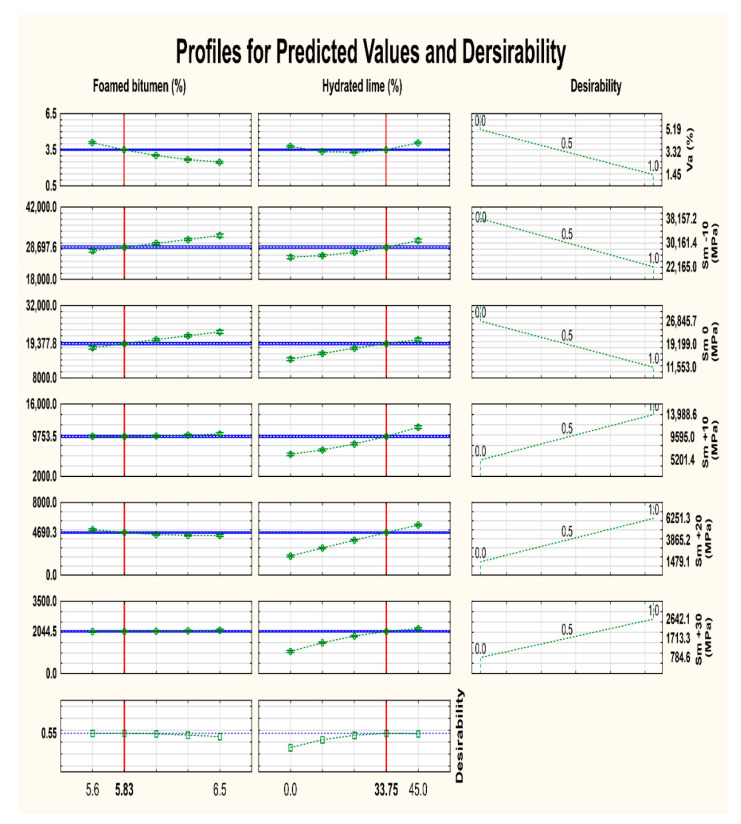
Profiles of approximated values and desirability function for the recommended amount of foamed bitumen and hydrated lime in asphalt concrete AC 8 S.

The performance of AC 8 S was measured using the following metrics: the most desirable parameter values were assigned the performance indicator equal to 1, and the least desired values were assigned 0. The used optimization procedure was described in detail in [75]. The following criteria were applied for the individual parameters of asphalt concrete:

Air void content V_a_ (max: 0, min: 1),
Stiffness modulus, S_m-10_, according to WT-2 (max: 0, min: 1),
Stiffness modulus, S_m0,_ according to WT-2 (max: 0, min: 1),
Stiffness modulus, S_m+10_, according to WT-2 (max: 1, min: 0),
Stiffness modulus, S_m+20_, according to WT-2 (max: 1, min: 0),
Stiffness modulus, S_m+30_, according to WT-2 (max: 1, min: 0),

The results of the optimization of hydrated lime and foamed bitumen in AC 8 S are shown in Figure 10.

The profiles of approximated values and the desirability of the adopted variables (asphalt concrete parameters) were analyzed, and the recommended content of foamed bitumen was determined at 5.8% and that of hydrated lime at 33.8%. Considering the process of dosing the components of the asphalt concrete and their tolerances, the following recommended amounts were adopted: 5.9% of foamed bitumen 50/70 containing 0.6% SAA and 30% of hydrated lime as a partial replacement for the mineral filler.

Assessment of the resistance of AC 8 S to permanent deformation was performed for the recommended content of foamed bitumen (5.9%) and hydrated lime at 0%, 15%, 30%, and 45%, as per WT-2 2014 [68]. According to the requirements of WT-2 2014, AC 8 S should have WTS_AIR_ < 0.15 and PRD_AIR_ < 9 [68].

**Figure 11:**
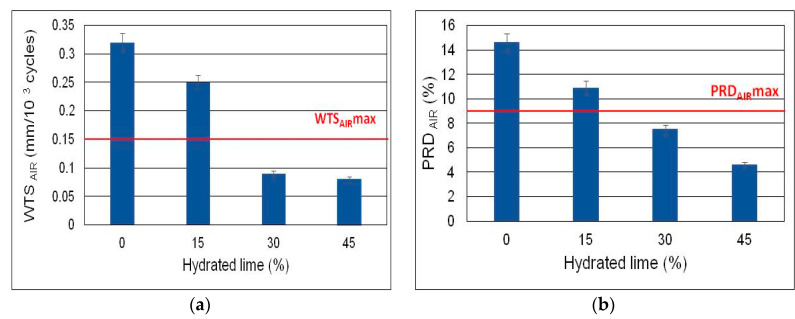
Resistance to permanent deformation of AC 8 S mixture at the recommended 5.9% content of foamed bitumen; (**a**) WTS_AIR_, (**b**) PRD_AIR_.

A graphical interpretation of the parameters representing the resistance of AC 8 S mixture to permanent deformation is shown in Figure 11.

Analysis of the results indicates that hydrated lime significantly influences the resistance to permanent deformation of HWMA mixture AC 8 S produced with foamed bitumen 50/70 modified with 0.6 wt% of SAA. Having 30% hydrated lime in its composition, AC 8 S meets the normative requirements, and is resistant to permanent deformation. Increasing the amount of hydrated lime to 40% leads to a slight increase in the value of the WTS_AIR_ parameter. A more significant effect of hydrated lime is observed in the PRD_AIR_ parameter. A 40% HL content affects the stiffness modulus at −10 °C more significantly than 30% HL content, which, as mentioned earlier, can promote low temperature cracking. For all these reasons, the 30% hydrated lime content, used as a partial replacement for the mineral filler, and the 5.9% foamed bitumen content (bitumen 50/70 treated with 0.6% SAA) were adopted as recommended in AC 8 S asphalt concrete.

The recommended composition in terms of the contents of foamed bitumen and hydrated lime ensures obtaining highly durable asphalt concrete AC 8 S, as shown by the significant resistance to moisture, frost, and permanent deformation.

## 4. Conclusions

Comprehensive analysis of the test data was the basis for the following conclusions concerning the HWMA mixture AC 8 S, produced with the addition of hydrated lime (HL):

- The content of air voids in AC 8 S shows an advantageous reduction with the addition of 15% hydrated lime (as a replacement of mineral filler) and at the content of SAA treated foamed bitumen of 5.6%. However, a higher HL percentage tends to increase the air void content in the mixture yet again. This may be because at higher concentrations, hydrated lime hinders asphalt concrete compaction. On the other hand, increasing the amount of binder has a positive effect on air void reduction in AC S 8, though the trend is the same as at 5.6%.
- The stiffness modulus tests conducted at −10 °C and 0 °C revealed an advantageous effect of hydrated lime, when used at 30% to 45%, and foamed bitumen 50/70 (modified with 0.6% SAA), when used at 5.9% to 6.5%. It was found that the interaction between the contents of the hydrated lime and foamed bitumen had a substantial influence on the stiffness modulus, as did the hydrated lime, used at 30% to 45%, at temperatures of 10 °C, 20 °C, and 30 °C.
- Optimization of HWMA concrete AC 8 S, in terms of its most relevant parameters allowed determining the optimum content of hydrated lime and foamed bitumen as 5.9% and 30%, respectively (dose tolerance considered).
- The asphalt concrete containing 5.9% bitumen 50/70 with 0.6% SAA, and hydrated lime content of 30% is characterized by high resistance to permanent deformation (WTS_AIR_ and PRD_AIR_), thereby ensuring the service durability of the pavement.
- The results of the tests revealed a significant effect of the SAA-modified (0.6%) foamed bitumen 50/70 and hydrated lime contents on the characteristics of HWMA mixture AC 8 S. The synergistic effect of the two materials is variable, and depends on the parameter analyzed.

The results of the stiffness modulus tests in the temperature range from −10 °C to 10 °C provide sufficient evidence to conclude that at temperatures below zero a AC 8 S-based pavement should perform well. No defects should appear. The high values of mechanical characteristics represented by the stiffness moduli at temperatures between +20 °C and +30 °C should ensure the resistance of the road surface to the effects of vehicle traffic. The values of WTS_AIR_ and PRD_AIR_ indicate a high resistance of AC 8 S to permanent deformation. This is particularly visible when hydrated lime content is 30%, for which the WTS_AIR_ reaches half the limit of this parameter. It follows from the above that HWMA concrete AC 8 S, produced with water-foamed bitumen and hydrated lime, can be recommended for road use (in situ) for a long-life deformation resistant asphalt concrete surface.

## Figures and Tables

**Table 1:** Properties of bitumen 50/70, and with 0.6% surface-active agent (SAA) content [29].

Property	Unit	Testing Method	Bitumen	
			50/700	50/70 + 0.6% SAA
Penetration at 25 °C	0.1 mm	PN-EN 1426	65.9	70.4
Softening point T_R&B_	°C	PN-EN 1427	50.4	48.8
Fraass breaking point	°C	PN-EN 12593	−15.1	−14.2
Temperature plasticity range	°C	-	65.5	63.0
Penetration Index	-	EN 12591	−0.6	1.4
Expansion ratio (ER)	-	-	11	19
Half-life t_1/2_	s	-	10	21
Foaming water content (FWC)	%	-	2.5	2.5

**Table 2:** Properties of limestone aggregates 0/2.

Property	Test	u.m.	Symbol
Dimension d/D	EN 933-1	-	0/2
Particle size distribution	EN 933-1	-	G_F_85
Density	EN 1097-6	Mg/m^3^	2.73

**Table 3:** Properties of gabbro aggregates 2/5 and 4/8.

Property	Test	u.m.	Symbol	
Dimension d/D	EN 933-1	-	2/5	4/8
Particle size distribution	EN 933-1	-	G_A_85	G_A_85
Density	EN 1097-6	Mg/m^3^	2.98	2.98
Shape index	EN 933-4	%	SI_20_	SI_15_
Flakiness index	EN 933-3	%	FI_20_	FI_15_
Percentage of crushed and broken surfaces	EN 933-5	%	C_90/3_	C_100/0_
Frost resistance	EN 1367-1	%	F_1_	F_1_
Abrasion resistance	EN1097-1	%	-	M_DE_15
Resistance to fragmentation	EN 1097-2	%	-	LA_15_

**Table 4:** Composition of AC 8 mineral mixture [72].

Materials	Mineral Mixture (% m/m)	Bituminous Mixture (% m/m)
Filler (limestone aggregate)	7.0	6.6
Crushed fine continuously graded aggregate 0/2 mm (limestone)	37.0	34.8
Coarse aggregate 2/5 mm (gabbro)	16.0	15.1
Coarse aggregate 4/8 mm (gabbro)	40.0	37.7
50/70 penetration paving-grade bitumen	-	5.6
Total	100.0	100.0

**Table 5:** Parameters of the model describing the relationships between V_a_ of AC 8 S and the contents of foamed bitumen with 0.6% SAA and hydrated lime.

Effect	Regression Coeff.	SE	*p*-Value	−95% Cnf. Lmt	+95% Cnf. Lmt
Variable: V_a_ (%), R^2^ = 0.880; R^2^ adj. = 0.875					
Intercept	68.159	6.791	<0.001	54.720	81.596
(1) Foamed bitumen (%)(L)	−18.425	2.247	<0.001	−22.872	−1.978
Foamed bitumen (%)(Q)	1.266	0.185	<0.001	0.898	1.6334
(2) Hydrated lime (%)(L)	−0.276	0.018	<0.001	−0.13	−0.241
Hydrated lime (%)(Q)	0.001	0.001	<0.001	<0.001	<0.001
1 L ∗ 2 L	0.038	0.003	<0.001	0.032	0.044

where Q = quadratic, L = linear.

**Table 6:** Stiffness modulus (S_m_) of the HWMA concrete, as a function of test temperature and the amount of foamed bitumen and hydrated lime.

Effect	Regression Coeff.	SE	*p*-Value	−95% Cnf. Lmt	+95% Cnf. Lmt
Variable: S_m-10_ (MPa), R^2^ = 0.8187; R^2^ adj = 0.8122, Pure Error MS = 1,809,712					
Intercept	31,976.28	45,578.49	0.4842	−58,208.5	122,161.1
(1) Foamed bitumen (%)(L)	−4250.82	15,082.22	0.7785	−34,093.6	25,591.9
Foamed bitumen (%)(Q)	535.14	1245.61	0.6682	−1929.5	2999.8
(2) Hydrated Lime (%)(L)	−560.66	122.82	<0.001	−803.7	−317.6
Hydrated Lime (%)(Q)	2.21	0.50	<0.001	1.2	3.2
1 L × 2 L	100.37	19.93	<0.001	60.9	139.8
Variable: S_m0_ (MPa); R^2^ = 0.8567; R^2^ adj = 0.8512; Pure Error = 1743,61					
Intercept	−14,876.7	44,742.85	0.7401	−103,408	73,654.63
(1) Foamed bitumen (%)(L)	6178.5	14,805.70	0.6771	−23,117	35,474.09
Foamed bitumen (%)(Q)	−203.2	1222.77	0.8683	−2623	2216.31
(2) Hydrated Lime (%)(L)	−178.1	120.57	0.1422	−417	60.52
Hydrated Lime (%)(Q)	−0.8	0.49	0.1111	−2	0.18
1 L × 2 L	61.4	19.56	<0.001	23	100.06
Variable: S_m10_ (MPa); R^2^ = 0.8714, R^2^ adj = 0.8667, Pure Error MS = 45,0536.1					
	42,843.7	22,741.54	0.0618	−2,154.3	87,841.71
(1) Foamed bitumen (%)(L)	−11,206.5	7525.32	0.1389	−26,096.6	3683.69
Foamed bitumen (%)(Q)	846.5	621.50	0.1756	−383.3	2076.24
(2) Hydrated Lime (%)(L)	−194.2	61.28	<0.001	−315.4	−72.92
Hydrated Lime (%)(Q)	1.2	0.25	<0.001	0.8	1.74
1 L × 2 L	43.8	9.94	<0.001	24.1	63.43
Variable: S_m20_ (MPa); R^2^ = 0.9434; R^2^ adj = 0.946; Pure Error MS = 79,096.06					
Intercept	44,108.5	9528.677	<0.001	25,254.4	62,962.65
(1) Foamed bitumen (%)(L)	−13,433.1	3153.101	<0.001	−19,672.1	−7194.16
Foamed bitumen (%)(Q)	1068.0	260.408	<0.001	552.7	1583.2
(2) Hydrated Lime (%)(L)	117.0	25.678	<0.001	66.2	167.82
Hydrated Lime (%)(Q)	−0.1	0.104	0.3932	−0.3	0.12
1 L × 2 L	−6.4	4.167	0.1282	−14.6	1.86
Variable: S_m30_ (MPa); R^2^ = 0.9285, R^2^ adj = 0.9259, Pure Error MS = 15,126.99					
Intercept	4073.402	4167.076	0.3303	−4171.87	12,318.67
(1) Foamed bitumen (%)(L)	−872.150	1378.912	0.5282	−3600.56	1856.26
Foamed bitumen (%)(Q)	61.340	113.881	0.5911	−163.99	286.67
(2) Hydrated Lime (%)(L)	6.551	11.229	0.5606	−15.67	28.77
Hydrated Lime (%)(Q)	−0.376	0.046	<0.001	−0.47	−0.29
1 L × 2 L	5.991	1.822	<0.001	2.39	9.60

**Table 7:** Correlations between AC 8 S parameters.

Variable	S_m-10_ (MPa)	S_m+30_ (MPa)	S_m+20_ (MPa)	S_m+10_ (MPa)	S_m0_ (MPa)	V_a_ (%)
S_m-10_ (MPa)	1.000	0.719	0.617	0.731	0.806	−0.086
	*p* = ---	*p* ≤ 0.001	*p* ≤ 0.001	*p* ≤ 0.001	*p* ≤ 0.001	*p* = 0.30
S_m+30_ (MPa)	0.719	1.000	0.912	0.847	0.756	0.191
	*p* ≤ 0.001	*p* = ---	*p* ≤ 0.001	*p* ≤ 0.001	*p* ≤ 0.001	*p* = 0.02
S_m+20_ (MPa)	0.617	0.9122	1.000	0.869	0.645	0.377
	*p* ≤ 0.001	*p* ≤ 0.001	*p* = ---	*p* ≤ 0.001	*p* ≤ 0.001	*p* ≤ 0.001
S_m+10_ (MPa)	0,731	0.847	0.869	1.000	0,696	0.347
	*p* ≤ 0.001	*p* ≤ 0.001	*p* ≤ 0.001	*p* = ---	*p* ≤ 0.001	*p* ≤ 0.001
S_m0_ (MPa)	0.805	0.756	0.645	0.696	1.000	−0.187
	*p* ≤ 0.001	*p* ≤ 0.001	*p* ≤ 0.001	*p* ≤ 0.001	*P* = ---	*p* = 0.02
V_a_ (%)	−0.086	0.191	0.377	0.347	−0.187	1.000
	*p* = 0.303	*p* = 0.022	*p* ≤ 0.001	*p* ≤ 0.001	*p* = 0.025	*p* = ---

Correlations and correlation coefficients are significant when *p* < 0.05.

**Table 8:** Parameters of the models characterizing the variables subjected to optimization.

Dependent Variable	SS Test for the Full Model with Respect to SS for Residual								
	Multicrit. R	Multicrit. R^2^	Adjusted. R^2^	SS Model	MS Model	SS Residual	MS Residual	F	*p*
V_a_ (%)	0.908	0.826	0.821	1.043 × 10^2^	26	22	0	165.1	<0.001
S_m-10_ (MPa)	0.888	0.789	0.783	1.224 × 10^9^	305,969,940	326,976,774	2,352,351	130.0	<0.001
S_m0_ (MPa)	0.920	0.846	0.842	1.435 × 10^9^	358,641,446	259,957,219	1,870,196	191.7	<0.001
S_m +10_ (MPa)	0.927	0.860	0.856	6.603 × 10^8^	165,078,470	107,443,778	772,977	213.5	<0.001
S_m+20_ (MPa)	0.971	0.942	0.941	2.325 × 10^8^	58,116,523	14,086,527	101,342	573.4	<0.001
S_m+30_ (MPa)	0.960	0.923	0.920	2.811 × 10^7^	7,028,122	2,340,397	16,837	417.4	<0.001
